# Epidemiological Association of Current Smoking Status with Hypertension and Obesity among Adults Including the Elderly in Korea: Multivariate Analysis of a Nationwide Cross-Sectional Study Excluding Grades 2–3 Hypertension Cases

**DOI:** 10.3390/jcdd11070212

**Published:** 2024-07-05

**Authors:** Sung-Eun Park, Seyong Jang, Wi-Young So, Junsu Kim

**Affiliations:** 1 Sport Medicine Major, Korea National Sport University, Seoul 05541, Republic of Korea; qkrtjddms2@naver.com; 2 Department of Taekwondo, College of Arts and Physical Education, Gachon University, Seongnam 13120, Republic of Korea; naganolala@gachon.ac.kr; 3 Sport Medicine Major, College of Humanities and Arts, Korea National University of Transportation, Chungju-si 27469, Republic of Korea; 4 Department of Sports and Outdoors, College of Bio Convergence, Eulji University, Seongnam 13135, Republic of Korea

**Keywords:** cross-sectional study, current smoking, epidemiology, hypertension, Korean population, multivariate analysis, obesity

## Abstract

Smoking is a major global health issue that contributes to various chronic diseases, while hypertension and obesity are considered significant health concerns due to their associated complications, such as cardiovascular diseases and metabolic disorders. In this study, we investigated the associations between current smoking status, hypertension, and obesity among the Korean population, excluding individuals with high blood pressure (systolic blood pressure ≥ 160 mmHg or diastolic blood pressure ≥ 100 mmHg) and those taking antihypertensive medications. Data from the 2015 Korea National Fitness Assessment, encompassing 3457 individuals, were analyzed. Logistic regression analysis was used to examine the effects of current smoking and other variables on hypertension and obesity. The results showed that, among the population that excludes specific hypertension criteria, current smoking status was not significantly associated with hypertension or obesity. However, sex and body mass index were significantly associated with hypertension, and age, sex, and blood pressure were significantly associated with obesity. Future research should utilize larger sample sizes and longitudinal designs to confirm these findings and include a broader range of hypertensive participants to better control for potential confounding variables.

## 1. Introduction

Smoking, as a worldwide habit, is considered a major risk factor for coronary artery disease, lung cancer, and stroke [1,2,3]. As a leading cause of death, it is also believed to be preventable through cessation [4]; however, smoking is difficult to quit because of its addictive nature. In fact, it has been reported that over 80% of smokers express a desire to quit, but only approximately 33% actually succeed, with approximately 80% returning to smoking within 6 months [5,6]. In 2015, the global prevalence of hypertension was estimated at approximately 1.13 billion, with a projected increase to 1.5 billion by 2025 and an expected annual death toll of 9.4 million due to complications such as heart disease, stroke, and kidney failure [7,8].

In 2017, smoking and increased systolic blood pressure were reported to be the highest risk factors for premature death in men worldwide [9]. Smoking, considered a major cause of cardiovascular disease, particularly for young adults, is associated with a significantly increased risk of cardiovascular disease later in life [10]. However, the association between smoking and hypertension has not been consistently reported. Some prospective cohort studies have reported smoking to be associated with an increased risk of developing hypertension [11,12], with older male smokers in the UK having higher systolic blood pressure than nonsmokers [13]. In a study in France, smoking was found to be a significant risk factor for hypertension in French men but not in French women [14]. However, other studies have shown that smokers have lower blood pressure than nonsmokers [15,16,17], indicating that smoking is not a risk factor for hypertension [18,19,20].

Besides smoking and hypertension, obesity is also a major public health concern worldwide. It is the fifth leading cause of global mortality, with approximately 800 million people classified as obese in 2016, and this is expected to reach approximately 1.12 billion by 2030 [21,22]. The life expectancy of smokers with obesity is approximately 13 years shorter than that of non-obese nonsmokers [23]. However, the relationship between smoking and obesity has also not been fully explored. The World Health Organization (WHO) has reported that regular smokers have a lower body mass index (BMI) than nonsmokers [24], and a cross-sectional study in the UK has reported a lower likelihood of obesity among smokers than among nonsmokers [25]. In contrast, other studies have reported no significant association between smoking and BMI [26,27]. Among Koreans, current smokers have a higher likelihood to have central obesity than nonsmokers, as evaluated by waist circumference [28].

Therefore, health issues, such as smoking, hypertension, and obesity, remain important global concerns, while the relationships among them remain unclear. Ongoing efforts to understand these relationships are crucial for addressing current public health issues. The prevalence of hypertension among Koreans has been steadily increasing, reflecting the aging population of Korea [29]. Additionally, among Korean adolescents, a consistent upward trend in the prevalence of hypertension has been observed, with an annual average change of 6.4%, particularly among those who are overweight or obese, indicating a higher risk of developing hypertension [30]. Similarly, obesity rates have been steadily increasing among Koreans, raising concerns about metabolic health issues, such as diabetes [31,32]. Both hypertension and obesity impose significant economic burdens on the country in terms of healthcare costs [33,34], and the increasing prevalence of these conditions suggests a growing economic burden. Furthermore, smoking remains an important public health issue in Korea. Despite ongoing anti-smoking campaigns, the smoking rate among Korean men remains high, with over 26.3% of males aged 15 and above reported as current smokers as of 2021 [35]. This high prevalence of smoking contributes to various health problems, including respiratory and cardiovascular diseases.

Therefore, in this study, we aimed to investigate the epidemiological associations between current smoking status, hypertension, and obesity in Korean men and women. By analyzing data from a representative national survey, we sought to understand how these factors are interrelated and identify the potential risk factors specific to the Korean population. It is crucial to explore these health issues by considering the unique characteristics and lifestyle factors of Korean men and women. The results of this study are expected to provide foundational data for developing public health strategies focused on the prevention and management of hypertension and obesity, complementing the existing research and guiding future health policies in Korea.

## 2. Materials and Methods

### 2.1. Participants

To analyze the relationships between current smoking status, hypertension, and obesity in Korea, the data from the 2015 Korea National Physical Fitness Survey were examined. The survey employed a multistage, stratified random sampling method based on sex, age, and region, using the Neyman allocation method to ensure representativeness. The appropriate sample size was determined, and samples were allocated by region, with the population including male and female adults aged ≥19 years nationwide, excluding Jeju Island. The measurements were conducted by local measurement agencies under the supervision of the main agency and collaborative research institutions [36].

The entire sample of the original survey included 3457 Korean individuals, with 1946 males and 1511 females. The age of all the participants in the survey ranged from 19 to 89 years, with the 89-year-old participants being present only in the male group. Their basic characteristics are presented in Table 1. The raw data did not include any identifiable information, such as name, phone number, home address, or resident registration number; thus, further ethical approval was not pursued. All research procedures were conducted under the control and approval of the Korea Institute of Sport Science and the Korea Ministry of Culture, Sports, and Tourism, adhering to the principles of the Helsinki Declaration.

### 2.2. Current Smoking Status

Participants were asked about their current smoking status through a questionnaire, where they selected one of three responses: currently smoking, smoked in the past but no longer smoking, or never smoked. Notably, the detailed information on smoking intensity and frequency was not provided. Therefore, participants were classified as smokers or nonsmokers based on their smoking status at the time of the survey, with former smokers included in the nonsmoking group.

### 2.3. Hypertension

Blood pressure was measured by nurses using a mercury sphygmomanometer (ALPK, Tokyo, Japan) and a stethoscope. Participants who visited the measurement agency for project participation wore the blood pressure cuff on their right upper arm after a 5 min rest in the seated position. Blood pressure was measured twice with a 2 min interval, and the average systolic blood pressure (SBP) and diastolic blood pressure (DBP) were recorded. The raw data used in this study excluded individuals who were taking antihypertensive medications and those with extremely high blood pressure (Grade 2–3 hypertension, SBP ≥ 160 mmHg or DBP ≥ 100 mmHg), as they had potential risks and limitations for participating in the various measurements [36]. The remaining participants were then classified as having Grade 1 hypertension (SBP ≥ 140 mmHg or DBP ≥ 90 mmHg) or normotensive based on the criteria defined by the American Heart Association [37] and the Korean Society of Hypertension [38]. The blood pressure measurement was conducted only once during the survey, which may not fully account for natural variations and potential measurement errors.

### 2.4. Obesity

BMI was used to assess obesity. It is calculated by dividing an individual’s weight (kg) by the square of their height (m) and is highly correlated with body fat percentage. The WHO Regional Office for the Western Pacific and the Korean Society for the Study of Obesity [39] define obesity as a BMI ≥ 25 kg/m^2^. This differs slightly from the criterion that classifies a BMI of ≥30 kg/m^2^ as indicative of obesity.

### 2.5. Breakfast Habits, Sleeping Hours, Exercise Frequency, and Stress Level

To analyze the relationships between current smoking status and hypertension and obesity, factors that can influence smoking, such as breakfast habits, sleep duration, exercise frequency, and stress levels, were explored. Participants were asked whether they regularly had breakfast, with response options of “regular breakfast”, “irregular breakfast”, “skipping breakfast daily”, and “replacing with snacks”. Those who reported having regular breakfast were classified as having a breakfast habit. Participants were also asked about their average daily sleep duration and the number of times per week they exercised for at least 30 min and broke a sweat. Additionally, participants were asked to rate their daily stress levels on a 5-point scale, and those who responded “very high” or “high” were classified as the high stress group, and those who responded “moderate”, “low”, or “very low” as the moderate stress group.

### 2.6. Procedure and Statistical Analysis

All data are presented as mean ± standard deviation or frequency (%). Mann–Whitney U tests were used to compare continuous variables between groups, and chi-square tests were applied for categorical variables. To evaluate the impact of current smoking on hypertension and obesity, we performed multivariate logistic regression analysis, adjusting for potential confounding factors such as age, sex, blood pressure, BMI, dietary habits (regular breakfast consumption), exercise frequency, sleep duration, and stress levels. The significance level for all statistical tests was set at *p* < 0.05. Statistical analyses were conducted using IBM SPSS Statistics for Windows, Version 26.0 (IBM Corp., Armonk, NY, USA).

## 3. Results

### 3.1. Effects of Current Smoking on Hypertension

The logistic regression analysis aimed to examine the impact of smoking on hypertension, excluding individuals with hypertension (SBP ≥ 160 mmHg or DBP ≥ 100 mmHg) and those taking antihypertensive medications. The results showed that smoking was not a significant predictor of hypertension among the remaining participants. Specifically, the odds ratio for hypertension in smokers, compared with that of nonsmokers, was not statistically significant (*p* = 0.731). This indicates that smoking status did not have a major influence on hypertension, particularly when participants with extremely high blood pressure or those taking antihypertensive medications were excluded. Age was also not a significant predictor of hypertension (*p* = 0.338). Regarding sex, females had a significantly lower probability of hypertension than males, with an odds ratio of 0.721 (*p* = 0.004). Exercise frequency did not significantly predict hypertension (*p* = 0.123), and regular breakfast consumption was also not significant (*p* = 0.555). Similarly, sleep duration was not a significant predictor of hypertension (*p* = 0.469), and high stress levels were not significantly associated with hypertension, compared with moderate stress levels (*p* = 0.236). However, BMI was an important predictor, with a 7.3% increase in the probability of hypertension for every one-unit increase in BMI (*p* < 0.001) (see Table 2).

### 3.2. Effects of Current Smoking on Obesity

The logistic regression analysis showed that, after excluding individuals with extremely high blood pressure and those taking antihypertensive medications, there was no significant association between current smoker (vs. nonsmoker) and obesity (*p* = 0.112, Exp (B) = 1.184). Age was a significant predictor, with each one-year increase in age associated with a 2.1% increase in the odds of obesity (*p* < 0.001, Exp (B) = 1.021). Women had significantly lower odds of obesity, compared with men, with an odds ratio of 0.428 (*p* < 0.001). Exercise frequency was not a significant predictor of obesity (*p* = 0.679, Exp (B) = 0.992) nor was regular breakfast consumption (*p* = 0.233, Exp (B) = 0.905). Sleep duration was also not a significant predictor (*p* = 0.130, Exp (B) = 0.948), and high stress levels were not significantly associated with obesity, compared with moderate stress (*p* = 0.281, Exp (B) = 1.107). However, both SBP and DBP were significant predictors of obesity. Each one-unit increase in SBP was associated with a 1.0% increase in the odds of obesity (*p* = 0.009, Exp (B) = 1.010), and each one-unit increase in DBP was associated with a 1.8% increase in the odds of obesity (*p* < 0.001, Exp (B) = 1.018) (see Table 3).

## 4. Discussion

In this study, we aimed to examine the relationships between current smoking status, hypertension, and obesity among Korean adults and older individuals, excluding individuals with high blood pressure (SBP ≥ 160 mmHg or DBP ≥ 100 mmHg) and those taking antihypertensive medications. Our research findings showed no significant associations between current smoking status and hypertension or obesity, but it is important to note that the generalizability of the results may be limited by the exclusion of certain hypertensive patients. Additionally, the non-significant associations observed in our study may be attributable to the cross-sectional design, which limited the ability to establish causal relationships. Furthermore, potential confounding factors, such as age-related health interventions and socioeconomic status, may have influenced the results.

However, these findings contrast with several previous studies that have confirmed the important associations between smoking and these health outcomes. For instance, Bowman et al. [11] and Halperin et al. [12] reported that smoking was significantly associated with an increased risk of hypertension over time. The cross-sectional design of our study may have limited the ability to detect such long-term effects, and the discrepancy in results may be due to the differences in research design. Cross-sectional studies provide a snapshot of data at a single point in time, which means they cannot determine the direction of relationships between variables or explain changes over time.

Furthermore, the varying outcomes may be attributed to differences in population demographics and health-related behaviors. For example, our study focused on the Korean population, which may have distinct lifestyle factors and genetic predispositions, compared with the populations explored in previous studies. The influence of cultural and social norms on smoking and health-related behaviors in Korea may also have contributed to the observed differences.

Potential confounding factors, such as age, socioeconomic status, and healthcare accessibility, may have also influenced the results. For instance, as people age, they generally have more frequent blood pressure monitoring, and smoking cessation is often recommended as an intervention for hypertension management with increased attention. This increased attention and medical intervention could act as confounding factors, potentially obscuring the direct effects of smoking on hypertension. Another potential confounding factor is socioeconomic status, which can influence health behaviors, access to healthcare, and overall health outcomes. This behavior may impact the associations between smoking, hypertension, and obesity. Therefore, future research should include socioeconomic variables to more effectively control these confounding factors.

Additionally, while the sample size of our study is substantial, it may not be large enough to detect more subtle associations or interactions between smoking, hypertension, and obesity. These limitations can impact the accuracy and generalizability of our findings. Therefore, the results of this study should be interpreted with caution, and future research should consider a longitudinal design and larger sample sizes to provide more definitive conclusions about causal relationships.

Previous longitudinal studies have examined the relationships between smoking, hypertension, and obesity over time, providing more robust evidence of causal pathways. For instance, some studies have shown that smoking cessation can lead to weight gain, which might complicate the relationship between smoking and hypertension due to the confounding effect of obesity [40,41]. In comparison, our study did not find a significant association between current smoking status and hypertension or obesity. This contrasts with some longitudinal studies that observed a higher incidence of hypertension among smokers over time [11,12]. For example, Bowman et al. found that women who smoked had an increased risk of developing hypertension over a 10-year period, and Halperin et al. reported a significant association between smoking and hypertension in middle-aged and older men, which were not consistent with our findings.

In this study, BMI and blood pressure were identified as significant predictors of hypertension and obesity, while smoking was not a significant factor. This is consistent with some longitudinal studies that have also reported non-significant associations between smoking and hypertension after adjusting for BMI and other covariates [16,17]. Additionally, our study found that females had a significantly lower probability of hypertension than males (*p* = 0.004), and for each unit increase in BMI, the odds of developing hypertension increased by 7.3% (*p* < 0.001). This aligns with general findings that higher BMI is a risk factor for hypertension.

Nicotine increases energy expenditure, suppresses appetite, and can mimic satisfaction derived from food [42,43]. Smoking one cigarette has been shown to increase energy expenditure by 3% within 30 min [44], while smoking four cigarettes can increase it by 3.3% within 3 h [45]. Many people, especially women and adolescents, believe that smoking can prevent obesity [46,47], and the effects of nicotine serve as evidence. In fact, a higher BMI in young women has been associated with smoking, and higher body dissatisfaction has been reported to increase the risk of smoking in both males and females [48]. Smoking initiated by such motivations can prompt overweight individuals to continue or increase their smoking [49]. From a physiological perspective, a higher BMI is associated with increased total blood volume and adipose tissue, which can lead to changes in metabolism, resulting in lower nicotine levels in the bloodstream for the same amount of smoking, potentially leading to increased smoking [49,50,51]. Additionally, the addictive nature of smoking can make quitting difficult or raise concerns regarding weight gain after quitting [25,52]. More than 80% of smokers express a desire to quit smoking; however, the actual percentage of those who successfully quit smoking is only approximately 33% [5,53]. Among those who successfully quit smoking, more than 80% experience weight gain [40] and 75–80% of quitters return to smoking within 6 months [6].

Previous studies conducted over a long period support the concept that smokers tend to have lower body weight and BMI than nonsmokers [41,54]. Despite these trends, it is important to note that smoking and obesity have a complex relationship influenced by factors such as sex, age, geographical characteristics, physical activity, dietary habits, mental health, and genetic traits [49]. Although a few studies have reported on the chronic metabolic effects of smoking, one study reported that the resting metabolic rate in women decreased by 16% after smoking cessation and remained unchanged until day 60. However, women who resumed smoking on day 30 experienced a 12% decrease in their resting metabolic rate, which returned to baseline by day 60 [55]. This study reported that weight gain occurred due to a decrease in the resting metabolic rate and an increased calorie intake. However, considering that physical activity increases the metabolic rate and that smokers tend to be less physically active than nonsmokers [56], the level of physical activity should be analyzed along with the relationship between smoking and obesity.

Some studies have indicated that weight gain in Caucasians is similar between smokers and nonsmokers, while others have shown that weight gain among African American smokers is lower than that in nonsmokers, implying that smoking does not always reflect a positive impact on weight control [57]. It has also been reported that excessive smoking increases the risk of obesity, while ex-smokers have a higher likelihood of obesity than current smokers and nonsmokers [25]. However, despite many studies confirming weight gain after smoking cessation, there can be individual differences in the amount of weight gain [49]. Additionally, because there may be a U-shaped relationship between smoking and BMI, the complexity of this relationship needs to be considered [55].

In this study, current smokers were defined as those who were smoking at the time of the survey. This limits our understanding of the nuanced relationship between current smoking status, hypertension, and obesity. Additionally, the lack of detailed information on smoking intensity and frequency may affect the accuracy of the research findings on smoking exposure. Future studies should consider not only current smoking status but also the quantity and duration of smoking to address the diversity of smoking behaviors. This should include the impact of previous smoking history and secondhand smoke exposure. Furthermore, self-reported data on smoking status may be subject to bias; hence, the use of objective measurements and biomarkers could help address this limitation. For example, a previous cross-sectional study of Koreans before 2013 found that abdominal obesity was more likely to occur in smokers based on waist circumference but not BMI [28]. Given the evidence that smoking is associated with abdominal obesity, fat distribution, and insulin resistance [58,59], incorporating additional obesity indicators, such as waist circumference, could strengthen the analysis. Exploring the relationship between smoking and diabetes could also provide additional insights into the metabolic consequences of smoking. The blood pressure measurements were conducted only once during the survey, which may not fully account for natural variations and potential measurement errors. Future research should consider integrating multiple measurements over time to obtain more reliable average values.

Given these limitations, future studies should consider including larger and more diverse samples of smokers, as well as a broader range of hypertensive participants. Additionally, the use of longitudinal research designs to track changes over time and the integration of various obesity and metabolic health indicators should be explored in future studies. Particularly, the cultural and social norms around smoking and dietary habits in the context of Korean society may influence health outcomes. Understanding these contextual factors could provide a more comprehensive understanding of the relationships between smoking, hypertension, and obesity. Additionally, expanding the scope of the investigation could contribute to more nuanced discussions on the health impacts of smoking and inform public health strategies to mitigate the adverse effects of smoking. The data used in this study are from 2015; hence, there may be limitations in interpreting and applying the findings to the current situation. While the relationships examined are likely still relevant, future research using more recent data would be necessary to confirm our findings.

## 5. Conclusions

This study specifically investigated the relationships between current smoking status, hypertension, and obesity among the Korean population, excluding individuals with high blood pressure (SBP ≥ 160 mmHg or DBP ≥ 100 mmHg) and those taking antihypertensive medications. The results showed no significant associations between active smoking and hypertension or obesity in this limited population. Our findings indicated that current smoking status was not significantly associated with hypertension or obesity, which is consistent with previous reports [16,17] of non-significant associations even after adjusting for confounding factors. However, this contrasts with the findings of prospective studies [11,12], which have indicated important associations in diverse populations. These discrepancies may be attributable to differences in study populations, research designs, measurement methods, and confounding control.

This study adopted a cross-sectional design to collect and analyze large-scale data within limited resources and time. While this was suitable for exploring the initial associations between current smoking status, hypertension, and obesity among Korean adults, the limitations of cross-sectional studies in establishing causal relationships prevented the study from making an appropriate contribution to scientific discussion. To address this, future research efforts should aim to elucidate the causal relationships between smoking, hypertension, and obesity through longitudinal study designs. Furthermore, including larger sample sizes and a broader range of hypertensive participants in future research would enhance the generalizability of the findings, allowing for a more detailed investigation into the complex relationships between smoking, hypertension, and obesity, and their implications for public health.

## Figures and Tables

**Table 1:** Physical characteristics of the participants.

Variables	Males (n = 1946)	Females (n = 1511)	*p*
Age (years)	42.77 ± 16.75	47.66 ± 18.79	<0.001 ***
Height (cm)	172.12 ± 6.51	158.53 ± 6.23	<0.001 ***
Weight (kg)	72.28 ± 9.77	56.91 ± 7.59	<0.001 ***
Systolic blood pressure (mmHg)	125.67 ± 11.54	122.17 ± 11.65	<0.001 ***
Diastolic blood pressure (mmHg)	77.05 ± 8.61	74.88 ± 9.15	<0.001 ***
Prevalence of hypertension (n, %)	303 (15.8%)	164 (11.0%)	<0.001 ***
Body mass index (kg/m^2^)	24.37 ± 2.81	22.68 ± 3.02	<0.001 ***
Prevalence of obesity (n, %)	720 (37.6%)	310 (20.8%)	<0.001 ***
Smoker (yes/no)	512 (26.7%)	31 (2.1%)	<0.001 ***
Breakfast (yes/no)	977 (51.0%)	813 (54.7%)	0.034 *
Sleeping hours (hours/day)	6.58 ± 1.08	6.61 ± 1.14	0.604
Exercise (frequency per week)	2.66 ± 2.01	2.56 ± 2.01	0.215
High stress level (yes/no)	451 (23.6%)	306 (20.6%)	0.039 *

Results are expressed as mean ± standard deviation or n (%). * *p* < 0.05 and *** *p* < 0.001, tested by Mann–Whitney U test and χ^2^ test.

**Table 2:** Effect of current smoking on hypertension.

Adults	Beta	S.E.	Wald	df	*p*	Exp (B)	95% C.I. for Exp (B)
Smoker (vs. nonsmoker)	−0.048	0.140	0.118	1	0.731	0.953	(0.725–1.254)
Age (years)	0.003	0.003	0.918	1	0.338	1.003	(0.997–1.009)
Sex (female vs. male)	−0.327	0.115	8.136	1	0.004 **	0.721	(0.576–0.903)
Body mass index (kg/m^2^)	0.070	0.017	16.745	1	<0.001 ***	1.073	(1.037–1.109)
Exercise (frequency per week)	−0.041	0.026	2.375	1	0.123	0.960	(0.912–1.011)
Breakfast (yes vs. no)	−0.064	0.108	0.349	1	0.555	0.938	(0.760–1.159)
Sleeping hours (hours/day)	0.033	0.046	0.525	1	0.469	1.034	(0.945–1.130)
Stress (high vs. moderate)	0.142	0.119	1.407	1	0.236	1.152	(0.912–1.456)
Constant	−3.626	0.538	45.365	1	<0.001 ***	0.027	

S.E.: standard error; df: degree of freedom; Exp (B): the odds ratio; C.I.: confidence interval. Note: the reference groups for the categorical variables are nonsmokers for current smoking status, males for sex, individuals who do not eat breakfast regularly for breakfast consumption, and those experiencing moderate stress for stress levels. ** *p* < 0.01 and *** *p* < 0.001, tested by logistic regression analysis.

**Table 3:** Effect of current smoking on obesity.

Adults	Beta	S.E.	Wald	df	*p*	Exp (B)	95% C.I. for Exp (B)
Smoker (vs. nonsmoker)	0.169	0.106	2.522	1	0.112	1.184	(0.961–1.458)
Age (years)	0.020	0.002	72.163	1	<0.001 ***	1.021	(1.016–1.025)
Sex (female vs. male)	−0.849	0.087	94.967	1	<0.001 ***	0.428	(0.361–0.508)
Systolic blood pressure (mmHg)	0.010	0.004	6.874	1	0.009 **	1.010	(1.002–1.017)
Diastolic blood pressure (mmHg)	0.018	0.005	12.900	1	<0.001 ***	1.018	(1.008–1.028)
Exercise (frequency per week)	−0.008	0.020	0.171	1	0.679	0.992	(0.953–1.032)
Breakfast (yes vs. no)	−0.099	0.083	1.422	1	0.233	0.905	(0.769–1.066)
Sleeping hours (hours/day)	−0.054	0.036	2.290	1	0.130	0.948	(0.884–1.016)
Stress (high vs. moderate)	0.102	0.094	1.164	1	0.281	1.107	(0.920–1.332)
Constant	−3.656	0.537	46.417	1	<0.001 ***	0.026	

S.E.: standard error; df: degree of freedom; Exp (B): the odds ratio; C.I.: confidence interval. Note: The reference groups for the categorical variables are nonsmokers for current smoking status, males for sex, individuals who do not eat breakfast regularly for breakfast consumption, and those experiencing moderate stress for stress levels. ** *p* < 0.01 and *** *p* < 0.001, tested by logistic regression analysis.

## Data Availability

The data presented in this study are available upon request from the corresponding author. The data are not publicly available because of the protection of personal information.
